# Determination of the mean duration of recent infection and false recency rate for the HIV triplex multiplex bead assay

**DOI:** 10.1371/journal.pone.0311829

**Published:** 2024-10-25

**Authors:** Robert A. Domaoal, Jeni Vuong, Amy Zheng, Mervi Detorio, Bharat S. Parekh, Ernest L. Yufenyuy

**Affiliations:** 1 Division of Global HIV & TB, Centers for Disease Control and Prevention, Atlanta, GA, United States of America; 2 Global Health Fellowship Program, Public Health Institute/Centers for Disease Control, Atlanta, GA, United States of America; Ethiopian Public Health Institute, ETHIOPIA

## Abstract

**Background:**

We developed the HIV Triplex multiplex bead assay to identify and serotype HIV infection with high sensitivity and specificity; and distinguish recent from long-term HIV-1 infections. It can facilitate accurate incidence estimation, while reducing the number of tests and blood collected, which is highly desirable for use in future studies and surveys. Using previously collected, treatment-naive longitudinal seroconversion HIV-1 positive panels and specimens from individuals infected for >12 months, we determined the assay’s mean duration of recent infection (MDRI) and false-recency rate (FRR) respectively, at various mean fluorescent intensity (MFI) cutoffs.

**Methods:**

We tested seroconversion specimens (N = 814) from 142 individuals infected with HIV-1 subtypes B, C, or AE, and 1341 cross-sectional specimens from individuals infected >12 months. The MFI cutoffs of 1000 to 2000 were evaluated for recency classification, including an MFI of 1250 corresponding to the limiting antigen avidity enzyme immunoassay (LAg-EIA) cutoff of 1.5 normalized optical density for MDRI and FRR. We used four statistical methods: Methods 1 and 2 used the empirically balanced observation time approach. Method 2 MFI values were raised to power = 1.33, based on a repeated measures model to linearize the relationship between MFI and time points, whereas Method 1 was not linearized. Methods 3 and 4 employed quadratic and linear interpolations for each seroconversion panel. FRR was calculated by dividing the number of specimens misclassified as recent by the total number of specimens tested.

**Results:**

MDRI values ranged from 135–146 days at MFI = 1000 to 229–279 days at MFI = 2000 by the 4 methods. FRR varied from 0.15%-1.27% with increasing MFI cutoff. At MFI = 1250, the average MDRI of 4 methods was 169 days and ranged from 159–183 with overlapping 95% CIs and FRR = 0.52%.

**Conclusion:**

The HIV Triplex assay demonstrates a longer dynamic range compared to current HIV recency assays with a low FRR for cutoffs examined. With a longer dynamic range and low FRR, the MDRI for recent infection can be extended as appropriate to detect more recent infections, increasing the value of incidence assays benefiting public health surveillance and future surveys.

## Introduction

Since the introduction of the UNAIDS fast track targets for eliminating the AIDS epidemic, many countries adopting these ambitious goals have reached or are nearing epidemic control [1]. On a global scale, new HIV infections decreased by 54% since 1996 and AIDS-related deaths by 68% since 2004 [2, 3]. While commitment has yielded much success at reducing new infections globally, progress has been slow in recent years, with inequities as major drivers of the ongoing epidemic [4]. As new cases are becoming harder to find, it is critical that data estimates are reliable and accurate to ensure appropriate and effective services and interventions.

Longitudinal surveillance and modeling methods were historically used to measure incidence estimates. However, use of biomarkers in laboratory-developed tests for recent HIV infection detection have largely replaced those methods for estimating national HIV incidence [5–9]. These laboratory-based tests can identify recent HIV infections based on the threshold used and the mean duration of recent infection (MDRI) of the assay. Current tests have set thresholds with MDRI’s that range from less than six months and up to one year. The LAg-EIA assay which has an MDRI of 130 days is the most widely used laboratory assay for incidence estimation and is widely accepted in HIV research, where testing is performed on samples that have been confirmed for HIV-1 positivity and serotyping. Therefore, HIV diagnosis, confirmation, and serotyping must be performed prior to LAg-EIA. Because external factors such as antiretroviral therapy, progression to AIDS and elite controllers can lead to inaccurate estimation of incidence results and affect recent infection detection, surveys have adopted a recent infection testing algorithm (RITA) [10–12]. The RITA adds viral load testing and ARV metabolite detection to LAg-EIA in addition to prior HIV diagnosis, confirmation and serotyping to improve the accuracy of recency classification, and hence incidence estimation in the presence of those external factors. As a result, an adequate sample volume is needed to ensure sufficient volume for all assays. An assay that can provide accurate incidence estimation, while reducing the number of tests and volume of blood collected, is highly desirable for use in future studies and surveys.

Previously, our laboratory developed and validated a bead-based HIV Triplex assay that can diagnose HIV infection, perform serotyping, and characterize a HIV-1 or HIV-1/2 infection as HIV-1 recent or long-term [13]. The test is highly sensitive and specific for both HIV-1 and HIV-2 diagnosis and has a high agreement with the LAg-EIA for recent and long-term characterization [13]. The test was further evaluated in the field using stored remnant specimens from the Nigeria AIDS Indicator & Impact Survey (NAIIS) [14]. The HIV Triplex assay results were similar for both diagnostic and recency characterization when compared to NAIIS HIV prevalence and incidence estimates. At the time of the field validation, the mean duration of recent infection (MDRI) for the HIV Triplex assay was inferred from correlation with the LAg-EIA where a cutoff of 1250 mean fluorescent intensity (MFI) for the HIV Triplex assay corresponded to a cutoff of 1.5 normalized optical density (ODn) for the LAg-EIA. The present study focuses on calculation of the MDRI using longitudinally collected seroconversion panels from participants infected with HIV and estimation of false recent rate (FRR) using specimens from individuals infected for >1 year. Robust statistical evaluations were used to finalize the assay specific MDRI and FRR parameters for accurate incidence estimation.

## Materials and methods

### Study population

Longitudinal seroconversion plasma specimens used in this study were previously collected from consenting individuals and characterized as part of various cohort studies in the Netherlands, Trinidad, Ethiopia, Thailand, and the United States [12, 15–20]. These specimens were previously characterized using HIV ELISA and Western blot (for HIV diagnosis), Multispot (for HIV-2 serotyping) and LAg-EIA (for recency classification), as appropriate [12, 21]. In total, 814 specimens from 142 seroconverting individuals were used to determine the mean duration of recent infection. Specimens were accessed for research beginning February 20, 2016. These specimens were obtained from different countries and comprise various subtypes: Amsterdam cohort (subtype B, n = 96), Ethiopia cohort (subtype C, n = 128), Trinidad cohort (subtype B, n = 48), Thailand Bangkok Metropolitan Administration (BMA) cohort (subtype B and AE, n = 485), Sero-Incidence Panel Project (SIPP) (subtype B, n = 57) [15–17, 19, 20, 22] (Table 1). Specimens from three studies collected from 1,341 individuals with HIV-1 infections greater than one year were used to estimate the FRR: HIV Epidemiologic Research Study (HERS) specimens (n = 261) from the U.S.; Thailand specimens (n = 128); and Ghana specimens (n = 952) [12, 18, 23].

**Table 1:** Panel composition of study population. Characteristics of seroconversion panels (A) and long-term HIV-infected specimens (B) used according to country of origin or study, specimens, and subtype.

A for MDRI determination			
**Country/Study**	**Subjects**	**Specimens**	**Subtype**
Amsterdam	24	96	B
Ethiopia	22	128	C
Trinidad	5	48	B
Thai BMA	85	485	B & AE
SIPP	6	57	B
**TOTAL MDRI**	**142**	**814**	
**B for FRR determination**			
**Country/Study**	**Subjects**	**Specimens**	**Subtype**
Ghana	952	952	CRF_AG, others
Thai	128	128	AE
USA (HERS)	261	261	B
**TOTAL FRR**	**1341**	**1341**	

Only specimens from treatment naïve individuals were used for determination of MDRI and FRR because early antiretroviral therapy (ART) affects the development and maturation of HIV antibodies and falsely elevates the FRR [24, 25].

This retrospective study was conducted under a protocol approved by the Centers for Disease Control and Prevention (CDC) Institutional Review Board (IRB) titled “Characterization, validation and application of new HIV-1 diagnostic tests and incidence assays to detect and diagnose recent HIV-1 infections and determine HIV-1 incidence estimates” (Project ID0900f3eb821d73a4). Only stored, unlinked, anonymous specimens collected under multiple CDC approved protocols working with individual countries (IRB # 5533, 5758) were used. Study was also approved by Bangkok Metropolitan Administration Ethics Committee, respective ministries of health and CDC. Ethical considerations of informed consent, privacy and confidentiality, beneficence and non-maleficence, transparency and reporting were reviewed, and local regulations and guidelines were followed. It was determined that this project does not require human subject research review because no contact with human subjects was involved, specimens were collected for another purpose, no extra specimens were collected, and identifying information was removed or not obtained so that data cannot be linked or re-linked with identifiable human subjects. Individuals donating the blood specimens provided written consent for use of the specimens for biological research. The data was analyzed anonymously, and the authors did not have access to any personal identifiable information that could identify individual participants during or after data collection.

### HIV triplex assay

Prior to testing the specimens, bead preparation, or coupling of the antigen to the magnetic beads was performed as previously described [13]. Briefly, the p24-gp41 fusion protein (Bioprocess, Inc., Australia) was coupled at saturating concentration (1 μg/1.5 million beads) on bead region 12 (Magplex Microspheres, Austin, TX) for diagnosis. Bead region 13 was coupled with the recombinant immunodominant region group M (rIDR-M) protein in limiting concentrations (0.04 μg/1.5 million beads) for the separation of recent and long-term infections while the HIV-2 immunodominant region (IDR) peptide was coupled at saturating amounts (10 μg/1.5 million beads) on bead region 14 for serotyping.

Testing was performed and results were interpreted as previously described [13].

Assay controls included on each plate consisted of a negative control tested in duplicate, and an HIV-1 positive (HIV-1) and HIV-2 positive (HIV-2) controls, both tested in triplicate. All samples were diluted 1:101 in assay buffer and plate(s) were incubated on a shaker for 60 minutes at room temperature, followed by incubation with phycoerythrin-conjugated goat anti-human antibody (Southern Biotech, Birmingham, AL) in assay buffer for 30 minutes and read on the MagPix instrument (Luminex, Austin, TX). The average MFIs acquired from at least 50 microspheres were used for the interpretation of the results for each sample and bead region.

### Determination of diagnostic sensitivity

All HIV positive specimens collected from the seroconversion panels and individuals with known long-term infections were used to calculate the diagnostic sensitivity by examining the MFI from bead region 12 of the HIV Triplex assay and determining the total number of specimens above and below the 4000 MFI cutoff. Sensitivity was calculated by (HIV POS (>4000 MFI))/ ((HIV POS (>4000 MFI)) + (HIV NEG (<4000 MFI))). Only HIV-1 or HIV-1/2 positive specimens were considered for the determination of the MDRI and FRR.

### Determination of the MDRI and FRR

The mean duration of recent infection (MDRI), the average time an individual is “recently infected” by the assay, was estimated using four different statistical methods that have been previously described [18]. Methods 1 and 2 are based on balancing both false recent and long-term rates within the first-year post seroconversion (e.g., “Empirically Balanced Observation Time” approach). This was based on early work done for incidence assays that indicate that false recency and false long-term rates must balance out, especially during the first year of post-seroconversion [11]. For Method 2, MFI values were raised to power λ = 1.33, as estimated using a repeated measures model, to linearize the relationship between MFI and time values. Daily values of MFI were then calculated by using linear interpolation for Methods 1 using the untransformed data and Method 2 using the transformed data. Confidence intervals were determined by 10,000 replicate MDRI estimates using specimen-level bootstrap resampling.

Method 3 calculated polynomial regression equations for each seroconversion panel (e.g., individual panel regression analysis). The midpoint between the last day the seroconversion panel tested negative and the first day the seroconversion panel tested positive was calculated and set as day 0. We then plotted each seroconversion panel in Excel which generated a regression equation. Using this equation we then solved for MFI cutoff to determine the number of days required to reach the MFI cutoff. The mean duration of recent infection was then calculated by calculating the mean of all individual results as well as a 95% confidence interval. The mean and confidence interval were calculated through 10,000 resampling iterations at the specimen-level with bootstrapped confidence intervals. Method 4 utilized a linear interpolation method where MFI values between visits were linearly interpolated for each specimen. Each specimen was assigned an MFI = 0 at infection (e.g., timepoint = 0) and no extrapolation was done beyond the last visit. Using the interpolated values the probability of testing “recent” recent at a specific timepoint is estimated by the proportion of specimens below the MFI threshold set at that timepoint. Suboptimal specimen panels that were missing specimens or specimen information, or that had less than 3 data points were excluded from the analysis as well as specimens that tested negative on the HIV Triplex assay.

Specimens collected from individuals with known long-term infection (>12 months) were used to calculate the FRR by taking those specimens misclassified as a recent HIV-1 infection by the HIV Triplex assay and dividing by the total number of specimens.

Both the MDRI and FRR were analyzed at MFI cutoffs of 1000, 1250, 1500, 1750, and 2000 with 95% confidence intervals. The MFI cutoffs were chosen above and below our inferred cutoff of 1250 that corresponded to a LAg cutoff 1.5 ODn with additional MFIs above 1250 to explore the possibility of extending the MDRI of the HIV Triplex assay. Individual seroconversion panel regression method using an MFI cutoff of 1250 was used for subtype comparison.

## Results

### Diagnostic sensitivity

We determined the assay diagnostic sensitivity for all 2155 specimens using previously defined cut off of 4000 MFI on bead 12 [13]. We found the diagnostic assay sensitivity to be 99.7% (2148 of 2155 were positive) (S1 Table) when compared to reference results generated using enzyme immunoassay (EIA) plus Western Blot algorithm. This suggests the HIV Triplex assay performs similar or better than current HIV-1 diagnostic assays. The assay specificity has been estimated in a previous study; it was not determined in this study because all samples tested were positive.

### Antibody avidity and kinetics

**Fig 1:**
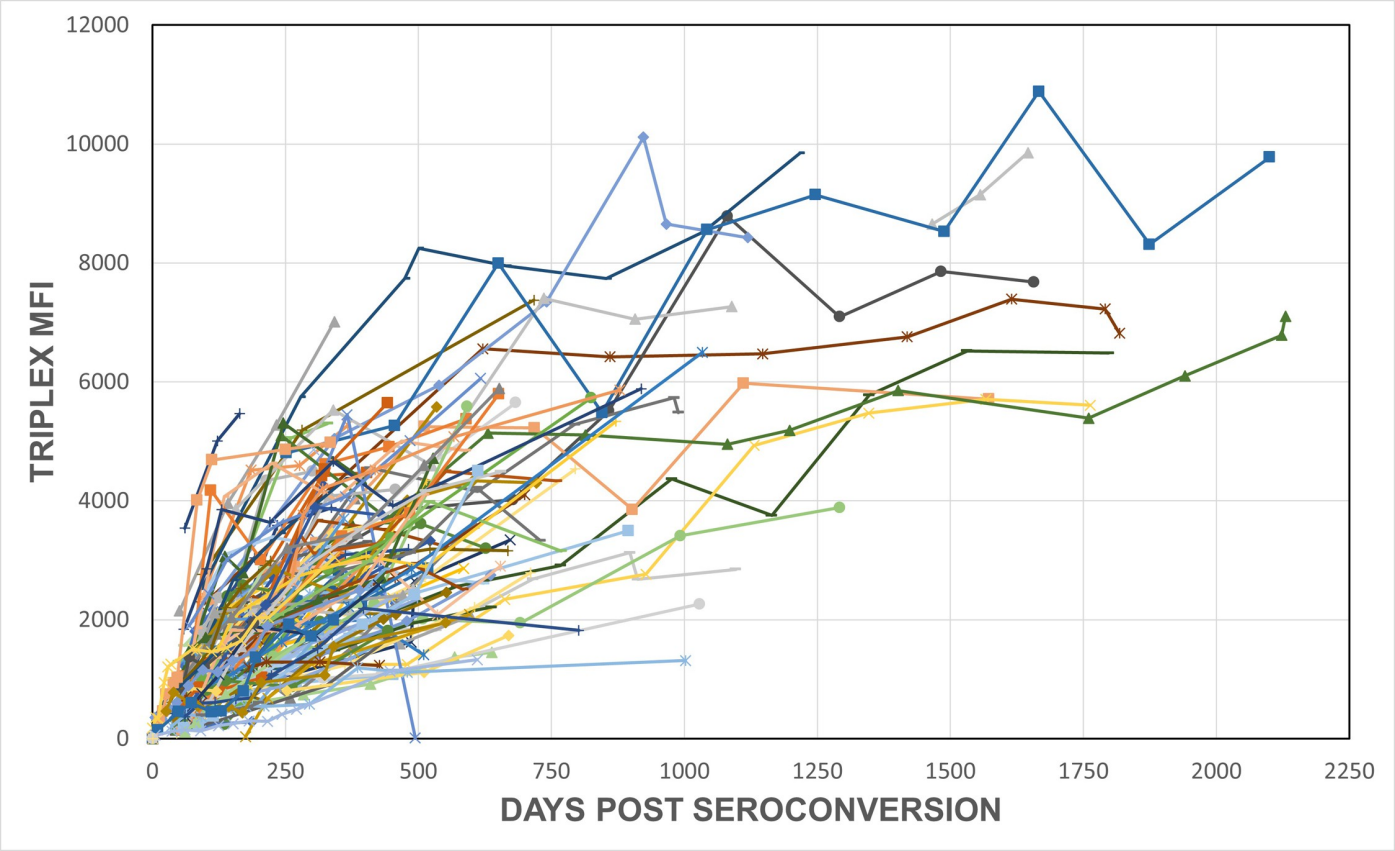
Antibody avidity measurements over time. Development of antibody avidity after seroconversion, as measured by the limiting concentration of rIDR-M (bead region 13) on the HIV Triplex assay for all 142 subjects; MFI is plotted versus days post seroconversion. Each line represents an individual subject.

A summary of the MFIs from all specimens collected post-seroconversion are plotted in Fig 1, with each line representing an individual and each dot representing time points when the specimens were collected from the individual. As expected, MFI values increased over time, demonstrating increasing avidity of the rIDR-M-specific antibodies following seroconversion.

### MDRI and FRR results

**Fig 2:**
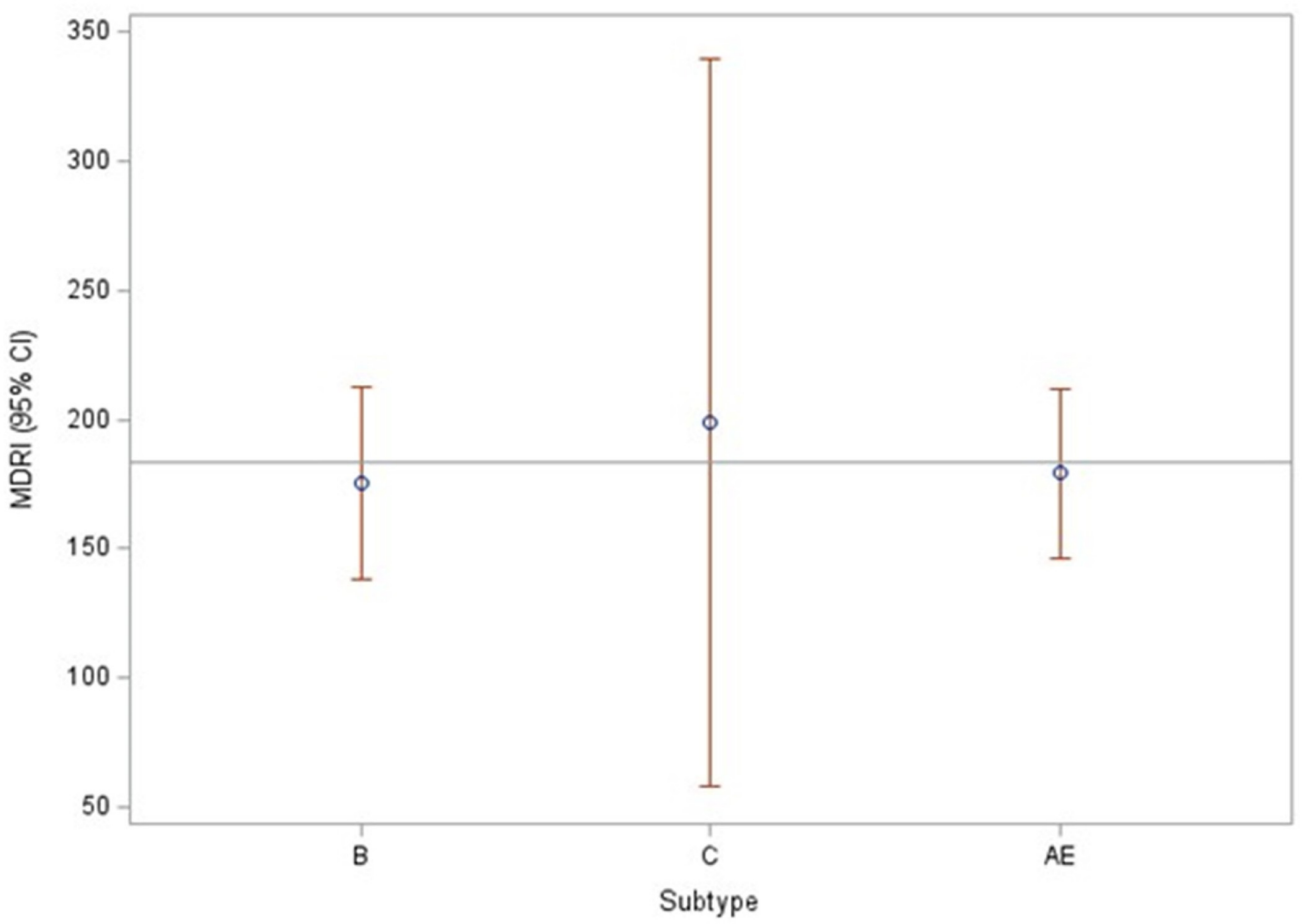
MDRI for three different HIV-1 subtypes at MFI cutoff of 1250 intensity units. The MDRI of HIV-1 subtypes B, C, and AE are shown for the samples tested. There were 218 subtype B samples, 128 subtype C samples and 68 subtype AE samples tested.

Results for the MDRI for each of the 4 statistical methods are described in Table 2. The overall average MDRI by 4 methods varied from 139 days to 243 days with increasing cutoffs corresponding to 1000 to 2000 MFI. While Method 1 yielded lower MDRIs and Method 3 yielded the highest values, minimal differences were observed among the different methods as demonstrated by the overlapping confidence intervals for each cutoff (Table 2). Since all methods produced similar MDRIs, MFI cutoff of 1,250 was selected for additional analysis according to subtype; the minimal difference in MDRI observed at each cutoff for each of the subtypes was not statistically significant (Fig 2). The FRR for each cutoff ranged from 0.15% to 1.27% (Table 2). Increases in MFI cutoffs corresponded to increases in both MDRI and FRR, as expected.

**Table 2:** MDRI and FRR values according to MFI cutoffs. Summary of 4 different statistical analyses used for determination of MDRI (in days, with 95% CI) at varying MFI cutoffs and corresponding FRR (%).

Cutoff MFI	1*	2	3	4	Average MDRI	FRR (%)
**1000**	139 (125, 153)	135 (121, 147)	146 (127, 165)	137 (108, 137)	139	0.15 (0.04–0.54)
**1250**	169 (152, 185)	167 (153, 182)	183 (158, 208)	159 (139, 179)	169	0.52 (0.25–1.07)
**1500**	193 (179, 206)	191 (177, 204)	209 (183, 234)	177 (157, 197)	192	0.82 (0.46–1.46)
**1750**	212 (198, 222)	209 (195, 220)	242 (214, 270)	198 (176, 220)	215	0.97 (0.57–165)
**2000**	229 (219, 238)	228 (218, 238)	279 (247, 310)	235 (208, 261)	243	1.27 (0.79–2.02)

*Methods 1 = Balanced time & 0,w and w,365, midpoint SC; 2 = Balanced time & 0,w and w,365, uniform SC; 3 = Individual SC Panel Regression; 4 = Linear Interpolation

## Discussion

Our data suggest that the HIV Triplex assay can serve as a promising assay for estimating incidence in cross sectional surveys while also providing results confirming HIV diagnosis and serotyping. We had previously shown the performance of the HIV Triplex assay in HIV diagnosis and serotyping and recent/long-term classification from in-house validation and field validation using NAIIS specimens [13, 14]. The HIV Triplex assay sensitivity of 99.7% found in this study agreed with previous findings [13].

We determined the MDRI and FRR for the HIV Triplex assay based on seroconversion panels and known long-term specimens from patients infected with HIV-1. Using 4 of the 7 statistical methods outlined in Duong et al. [18], our results also revealed minimal differences in MDRI calculations among the 4 methods at each cutoff (Table 2). The overlapping CIs among the methods and the narrow MDRI range (4 to 9 months) for all the different cutoffs overall suggest robust assay results.

Determination of a cutoff for an assay requires balance between the MDRI and the FRR. As the cutoffs increase, both MDRI and FRR increase accordingly. We found that at 1250 MFI cutoff, the MDRI ranged from 159 days to 183 days (average 169.5 days) with a corresponding low FRR of 0.52%. For comparison, this is less than 1.3% FRR at the 1.5 ODn cutoff for the widely used LAg assay when the MDRI was determined using similar test characteristics and sample set [18]. The HIV Triplex assay could extend the MDRI used in comparison with the LAg-EIA assay by about 2 months with no significant increase in false recency rates (Table 2). Except for MFI 2000, all other cutoffs yielded low mean FRRs of <1%. This is an improvement over the LAg-EIA assay where higher FRRs were observed for cut-offs greater than 1.5 ODn, probably due to a narrower dynamic range [25]. The improved low FRR associated with increased MDRI accorded by the HIV Triplex assay may be attributed to the wider dynamic range with MFIs ranging from single digits to tens of thousands. When compared to other incidence assays, the HIV Triplex assay at MFI 1250 cutoff performs better than most of the assays that have been previously evaluated by CEPHIA [26]. Additionally, because of the heterogeneity in LAg assay thresholds, there is a need for context-specific test characteristics, like subtype, population and algorithms when using LAg as a reference [27]. Hence, the current study used only limited number of HIV-1 subtypes and these findings may not be generalized to all other subtypes especially subtypes A and D that have traditionally used different MDRI in surveys that have used LAg assay [28]. The target product profile (TPP) for recency assays as agreed upon by the WHO Technical Working Group on HIV Incidence Assays, a group of experts working on incidence assays, recommend a longer MDRI to capture a larger number of recent infections in a population [29]. The HIV Triplex assay’s wide dynamic range suggests that this assay fits this TPP. Even at the highest cutoff of 2000, the FRR is 1.27%, which is lower than what has been observed in most assays evaluated to date [18, 30].

Our analysis has important limitations. Although we tested samples from 6 countries and three different subtypes, more seroconversion panels from different HIV subtypes such as subtype A and D are still needed to fully evaluate the performance of this assay on those subtypes which might affect the MDRI. Our findings may not be generalizable to all other subtypes and larger studies are still needed. However, like previous concerns about assays relying on the maturation of antibody response, the addition of viral load and drug metabolites in the testing algorithm are also needed to improve on the positive predictive value of recent infection and hence incidence estimates. Although the Triplex assay will reduce the amount of sample needed for testing, the time needed for multiple tests, and the cost for multiple tests, samples still would require additional testing in the RITA algorithm to control for virally suppressed repeat testers on ARV and increase the accuracy of recency classification. Ideally, biomarkers that are not affected by treatment would be highly desirable. We are currently exploring aptamer technology and other approaches for the identification of new biomarkers for HIV infection and recent infection detection that are not affected by treatment or pre-exposure prophylaxis (PrEP) [31]. Further studies are also needed to evaluate the overall efficiency and cost-effectiveness of using the HIV triplex assay compared to current individual methods.

A high performing incidence assay should be robust to account for subtype differences. Sub-type analyses were conducted and were similar for B, C, and AE, however sub-type C had a larger confidence interval which may be due to more variability (Fig 2). Data from the subtypes performed as expected since the biomarker utilized in the Triplex assay is a multi-subtype recombinant antigen designed from the immunodominant region of gp41 from group M viruses and has been shown to perform similarly across different subtypes [17, 21]. However, testing on additional subtypes especially subtypes AE and D that have not been previously tested is needed to assess whether a weighted average or sub-type specific MDRI is warranted in mixed subtype populations. Field evaluations are needed in multiple countries and in different program settings to assess the performance of this assay and its potential use in HIV prevention programs.

## Conclusion

Assays for determination of recent infection, particularly those with a low FRR and MDRI close to one year, are highly desirable for HIV-1 incidence estimates in cross-sectional surveys. We found that the HIV Triplex assay has a longer MDRI of 159 to 183 days (average 169.5 days) with a corresponding low FRR of 0.52 at a 1250 MFI cutoff. The assay also has a potential to extend the MDRI without significant increase in FRR. These results demonstrate that the bead-based HIV Triplex assay can be used in place of the current assays due to its low FRR and longer MDRI range, allowing for more detection of recent infections. Moreover, because the assay can provide HIV confirmation and typing information concurrently, this test can help to reduce the number of tests performed for HIV diagnosis confirmation and serotyping thereby streamlining the overall processes of HIV testing (number of tests required, volume of sample, equipment, and technicians etc.), at potentially lower cost with remarkable data quality. The HIV Triplex assay therefore offers a path for more frequent and sustainable HIV prevalence and incidence surveillance.

## Supporting information

S1 Table Assay diagnostic sensitivity. This table shows the assay diagnostic sensitivity for the HIV Triplex assay diagnostic Bead 12 compared to the HIV status determined by EIA plus Western Blot.
(DOCX)

S1 Data Study data set. (XLSX)
