# Supplementary material for: Determination of the mean duration of recent infection and false recency rate for the HIV triplex multiplex bead assay
Source: PLoS One. 2024 Oct 25;19(10):e0311829. doi: 10.1371/journal.pone.0311829 (PMC11508083; doi:10.1371/journal.pone.0311829)
Supplement: S1 Table — This table shows the assay diagnostic sensitivity for the HIV Triplex assay diagnostic Bead 12 compared to the HIV status determined by EIA plus Western Blot. (DOCX) [file pone.0311829.s001.docx]

**S1 Table. Assay Diagnostic Sensitivity.**

|  | **HIV Status (EIA plus Western Blot)** | | | |
| --- | --- | --- | --- | --- |
|  |  | **HIV Positive** | **HIV Negative** | **Total** |
| **HIV Triplex Assay Bead 12** | **HIV Positive (>4000 MFI)** | 2148 | 0 | 2148 |
|  | **HIV Negative (<4000 MFI)** | 7 | 0 | 7 |
|  | **Total** | 2155 | 0 | 2155 |
|  |  |  |  |  |
|  | **Sensitivity** | **99.7 %** |  |  |
|  | **Positive Predictive Value** | **100 %** |  |  |
|  | **Negative Predictive Value** | **0 %** |  |  |
